# Autosomal dominant polycystic kidney disease with ectopic unilateral multicystic kidney: a case report

**DOI:** 10.1186/s13256-023-04305-1

**Published:** 2024-01-09

**Authors:** Yaw Amoah, Mathew Yamoah Kyei, James Edward Mensah, Bridget Palm, Henry Kwasi Adrah, Isaac Asiedu

**Affiliations:** 1 Urology Unit, Department of Surgery, Korle-Bu Teaching Hospital, Accra, Ghana; 2 Department of Surgery, University of Ghana Medical School, Accra, Ghana; 3 Department of Radiology, Korle-Bu Teaching Hospital, Accra, Ghana

**Keywords:** Autosomal dominant polycystic kidney disease, Ectopic kidney, Renal function

## Abstract

**Background:**

Autosomal dominant polycystic kidney disease (ADPKD) is the most common hereditary renal disorder and the fourth cause of death of end-stage renal disease. The disease has a prevalence of 1:400–1:1000 accounting for 10% of patients on dialysis. In most ADPKD patients, bilateral kidneys are similarly affected, with numerous fluid-filled cysts arising from different nephron segments. Only a few cases of ADPKD with ectopic unilateral multicystic kidney have been reported. It has been observed that the deterioration of their kidney function seemed to be quicker than their age- and sex-matched controls and siblings especially when the ectopic kidney is dysplastic.

**Case presentation:**

We report a case of a 46-year-old Ghanaian male patient who presented with left flank pain and hematuria with high BP and deranged renal function. Abdominal ultrasonography showed both kidneys to be larger than normal and had multiple cysts of varying sizes with the right kidney located in the right iliac fossa. Follow up Abdominopelvic computer tomographic scan (CT–Scan) without contrast showed enlarged kidneys with the renal parenchyma replaced by innumerable cyst of varying sizes. The right kidney was ectopically located in the right aspect of the pelvis. A diagnosis of ADPKD with right pelvic ectopic multicystic kidney was made. He was put on antihypertensives, analgesia for the left flank pain and to have follow up at the urology and nephrology departments.

**Conclusion:**

In most ADPKD patients, bilateral kidneys are similarly affected. Only a few cases of ADPKD with ectopic unilateral multicystic kidney have been reported. It has been observed that the deterioration of their kidney function seemed to be quicker than their age- and sex-matched controls and siblings especially when the ectopic kidney is dysplastic.

## Background

Autosomal dominant polycystic kidney disease (ADPKD) is the most common hereditary renal disorder and the fourth cause of death of end-stage renal disease. The disease has a prevalence of 1:400–1:1000 accounting for 10% of patients on dialysis [1].

The disease is caused by mutations in the *PKD1* (85% of cases) or *PKD2* (15% of cases). In most ADPKD patients, bilateral kidneys are similarly affected, with numerous fluid-filled cysts arising from different nephron segments. Only a few cases of ADPKD with ectopic unilateral multicystic kidney have been reported [2]. It has been observed that the deterioration of their kidney function seemed to be quicker than their age- and sex-matched controls and siblings especially when the ectopic kidney is dysplastic [3].

We report a case of ADPKD patient with right pelvic ectopic multicystic kidney who presented with left flank pain and hematuria and current literature on the clinical implication of this associated relatively rare finding. This work/case has been reported in line with the CARE guidelines [4].

## Case report

A 46-year-old Ghanaian male presented to our outpatient department (OPD) with a history of hematuria and left flank pain. The hematuria was painless and total. The pain was dull in nature; constant, non-radiating with no aggravating or relieving factors. There were no lower urinary tract symptoms and no associated fever or vomiting. There was no childhood history of hematuria. His blood pressure was 154/112 mmhg. There was no abdominal tenderness or mass on examination. Other physical examinations were unremarkable.

The full blood count had hemoglobin of 13.2 g/dl. His renal function was deranged. [sodium 140 mmol/l, potassium 4.9 mmol/l, urea 13.6 μmol/l, creatinine 518 μmol/l, eGFR 13 ml/min].

**Fig. 1:**
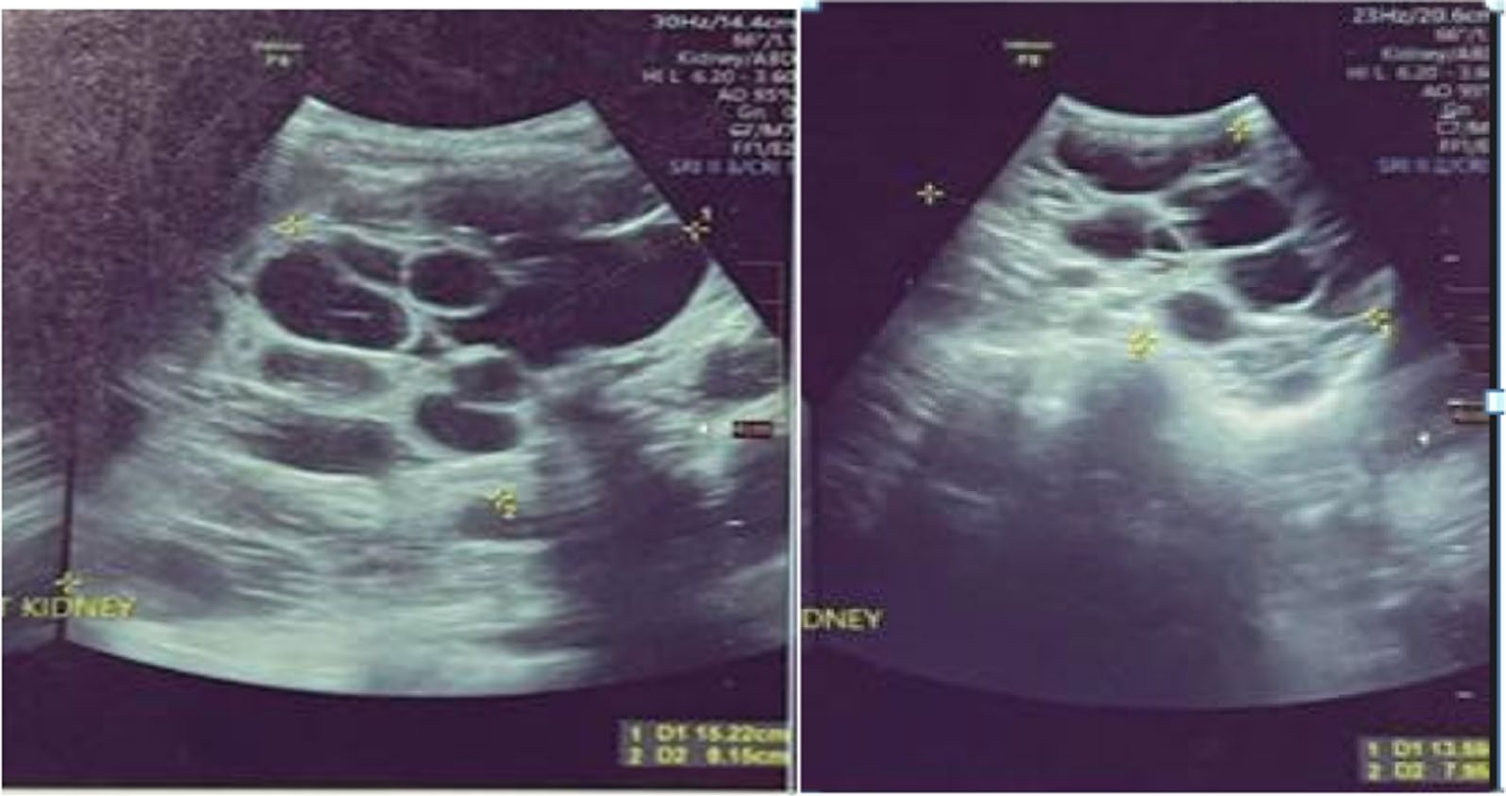
Polycystic kidney on Ultrasound

Abdominal ultrasonography showed both kidneys to be larger than normal and had multiple cysts of varying sizes with the right kidney located in the right iliac fossa (Fig. 1).

**Fig. 2:**
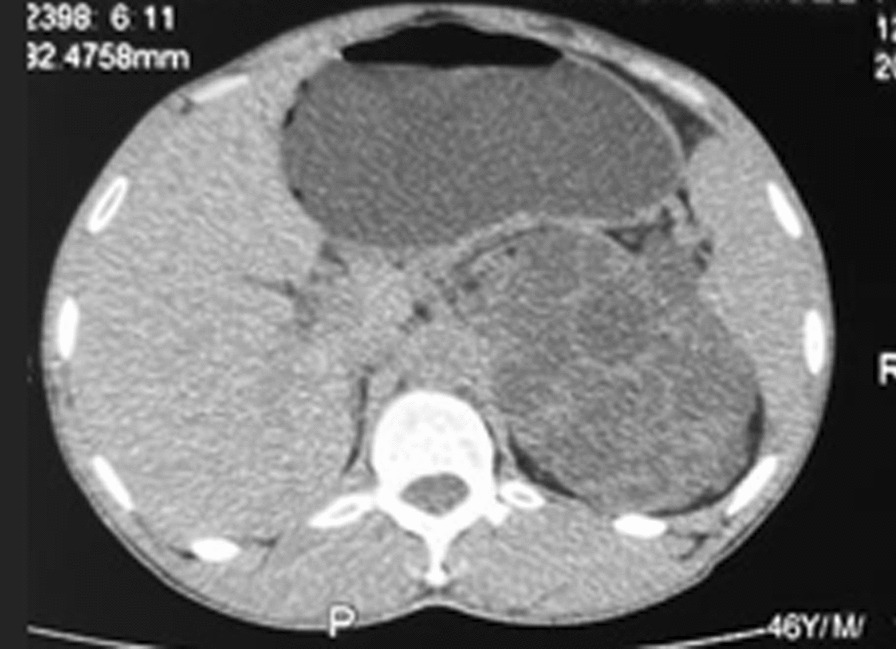
Left orthotopic polycystic kidney disease on CT—Scan (transverse view)

**Fig. 3:**
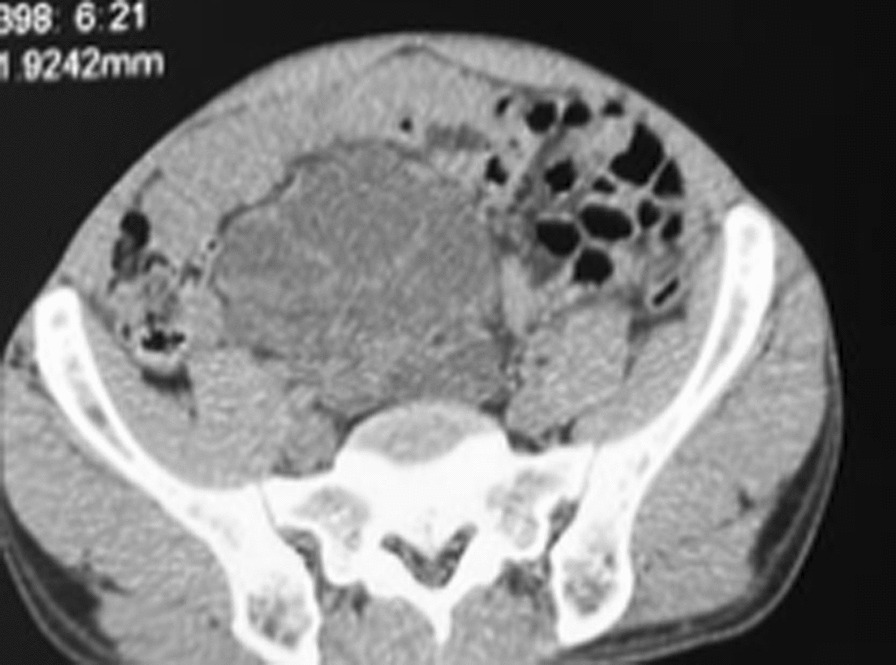
Right ectopic polycystic kidney disease on CT—Scan (transverse view)

**Fig. 4:**
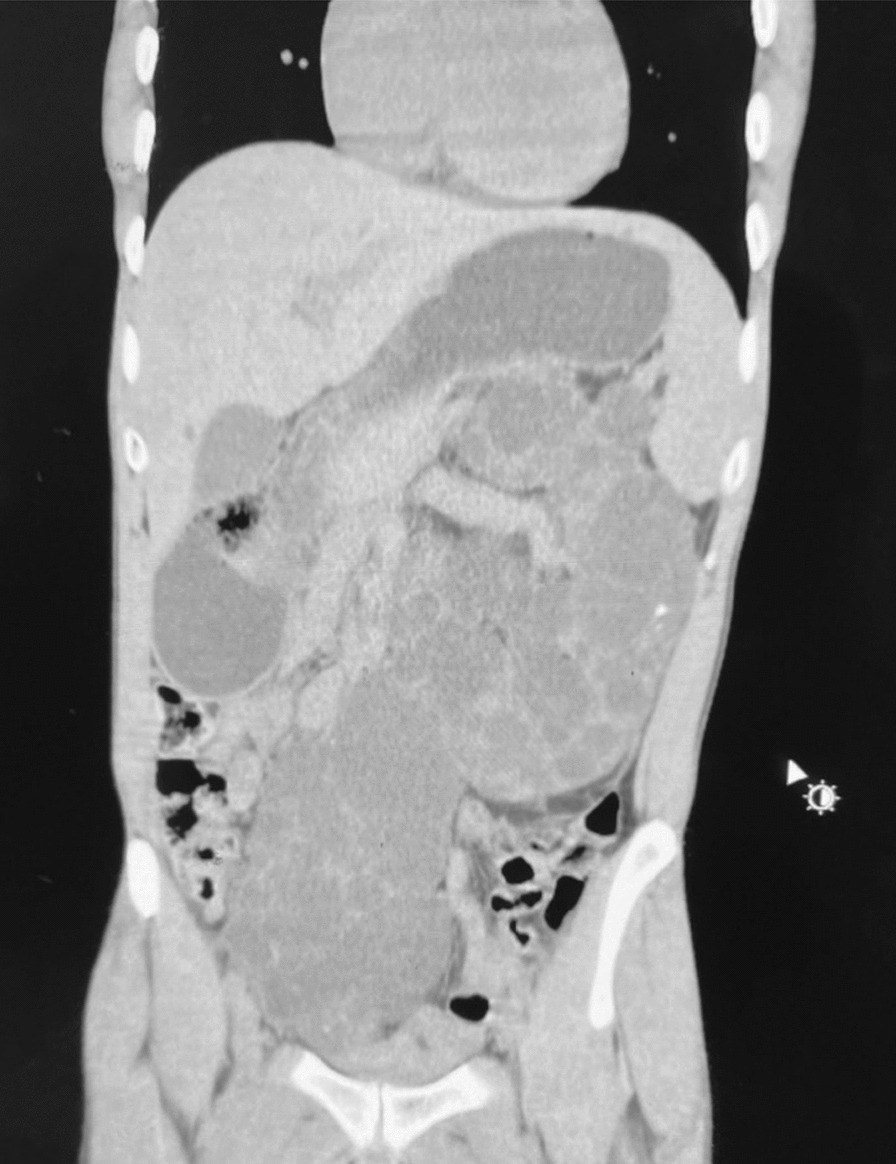
Right ectopic and left orthotopic polycystic kidney disease on CT—Scan (Coronal view)

Abdominopelvic computer tomographic scan (CT-Scan) without contrast showed enlarged kidneys with the renal parenchyma replaced by innumerable cyst of varying sizes. The right kidney was ectopically located in the right aspect of the pelvis. The right and left kidneys measure 18.8 cm × 8.0 cm and 21 cm × 9.8 cm respectively. The cysts were hypodense with thin walls with some of the walls calcified. The other intra-abdominal organs did not have cysts (Figs. 2, 3 and 4).

A urine cytology was negative for malignancy and urethrocystoscopy showed normal findings with no lateralizing signs. Renal scintigraphy was not done but will be considered, if necessary, in subsequent follow ups. The symptoms were hence attributed to the polycystic disease.

He was put on antihypertensives, analgesia for the left flank pain and to have follow up at the urology and nephrology departments.

## Discussion

ADPKD is a relatively common disease with a prevalence of 1:400–1:1000 and is associated with up to 10% of end-stage renal disease patients on dialysis. A minority of patients may go undiagnosed throughout their lifetime due to age-dependent penetrance and variable expressivity [1]. Although there is extensive allele heterogeneity, the ADPKD causing mutations are found in the PKD1 locus (chromosome 16 abnormality (16.p13.3); 85% of cases), the PKD2 locus (chromosome 4 abnormality (4p21); 15% of cases), and a rarer and newly discovered third locus, GANAB (chromosome 11 abnormality (11q12.3); 0.3% of cases); the GANAB locus is also associated with autosomal dominant polycystic liver disease [5].

The most common clinical presentation is a urinary tract infection [1]. Flank or abdominal pain is also a common presenting symptom and may be due to renal capsule expansion, renal pedicle traction, compression of adjacent abdominal organs, nephrolithiasis, renal hemorrhage and chronic urinary tract infections [1]. Our patient had a left flank pain but no pain associated with the right ectopic kidney.

Gross hematuria is common clinical presentation in autosomal dominant polycystic kidney disease (ADPKD). Cyst hemorrhage, cyst infection, urinary tract infection, renal stones and underlining malignancy are frequent cause of hematuria in ADPKD [6].

Typically, renal failure occurs by the fourth to sixth decade of life although the onset can be variable [7]. Our patient had derangement of the renal function with eGFR of 13 ml/minute (chronic kidney disease stage 5).

Renal ectopy results from the disruption of the normal embryological migration of the kidneys from the pelvis to the retroperitoneal renal fossa (at the level of the second lumbar vertebra). It may result from abnormalities of the ureteral bud and metanephric blastema, genetic anomalies, teratogenic influences or from anomalous vasculature acting as a barrier to ascent. The incidence of pelvic kidney is 1 in 5000 [8]. Incidence of ectopic autosomal dominant polycystic kidney disease is not available. Very few cases have been reported in the literature. Gupta *et al.* published the last case in 2014 [9]. Other case reports on autosomal dominant polycystic kidney disease with ectopic kidney had been previously published by Connor *et al.* in 2009 [8], Chen *et al.* in 2010 [10], Solak *et al.* in 2012 [11] and Xu *et al.* in 2013 [3]. Thus, this case is a rare finding worthy of reporting.

The clinical importance of this rare condition stems from the possibility of aberrant vascular structures and need for preparation of an appropriate place for grafting kidney during renal transplantation. Ectopic pelvic kidneys may accompany iliac arterial abnormalities and this should be evaluated before transplant surgery [11].

Disease progression with the ectopic ADPKD is observed at a significantly lower age [4] Our patient at 46 years had a stage 5 chronic kidney disease making him a candidate for renal transplantation.

Diagnosis relies on imaging with typical findings of enlarged kidneys containing multiple cysts. Although less reliable in younger patients (less than 18 years), screening renal ultrasonography should be considered in asymptomatic patients with an ADPKD family history; moreover, some centers mandate genetic testing for a definitive diagnosis [6]. Genetic testing was not done for the case presented as we didn’t have this facility at our center and hence the diagnosis was based on findings on CT scan imaging.

Other extrarenal manifestations of ADPKD include hypertension (occurring in 60% of patients with ADPKD prior to renal function impairment), cerebral aneurysms, hepatic cysts, pancreatic cysts, cardiac valvular disease (mitral, aortic and tricuspid insufficiency), aortic root dilation, colonic diverticula, abdominal wall and inguinal hernias, and seminal vesicle cysts [1, 3]. Lastly, there is a reported association between RCC and ADPKD. RCC may present as a cystic renal mass, and a small percentage of electively excised polycystic kidneys are found to contain a malignant neoplasm. RCC should be considered if a complex cyst (defined as a cyst with calcifications, septations or wall thickening) grows rapidly or is associated with symptoms that are inconsistent with the underlying disease severity [7]. Although some calcifications were seen on the imaging these were isolated and also the urine cytology was negative for malignant cells. Thus, renal cell carcinoma was considered excluded but patient is to have follow up. Vigilance during the workup of such polycystic masses for RCC is important [12].

## Conclusion

Autosomal dominant kidney disease with ectopic kidneys is rare. The clinical findings are as seen with Polycystic Kidneys Disease with orthotopic kidneys; however, it has been observed that the deterioration of their kidney function progression is observed at a relatively younger age. The possibility of associated aberrant vascular structures and iliac arterial abnormalities require meticulous evaluation to help choose an appropriate place before graft placement at renal transplantation.

## Data Availability

Not applicable.

## Abbreviations

ADPKD: Autosomal dominant polycystic kidney disease
CT scan: Computer tomographic scan
RCC: Renal cell carcinoma
eGFR: Estimated glomerular filtration rate

## References

[1] Silverman J, Desai C, Lerma EV (2015). Autosomal dominant polycystic kidney disease. Dis Mon.

[2] Cornec-Le Gall E, Torres VE, Harris PC (2009). Cardiovascular abnormalities in autosomal-dominant polycystic kidney disease. Nat Rev Nephrol.

[3] Xu J, Chen DP, Mao ZG (2013). Autosomal dominant polycystic kidney disease with ectopic unilateral multicystic dysplastic kidney. BMC Nephrol.

[4] Gagnier JJ, Kienle G, Altman DG, Moher D, Sox H, Riley D (2013). The CARE guidelines: consensus-based clinical case reporting guideline development. J Med Case Rep.

[5] Gerstein NS (2018). Severe autosomal dominant polycystic kidney disease. BMJ Case Rep.

[6] Bhavna B, Mohammed A (2018). Juan valuation and management of gross hematuria in autosomal dominant polycystic kidney disease: a point of care guide for practicing internists. Am J Med Sci.

[7] Ecder T, Schrier RW (2018). Genetic complexity of autosomal dominant polycystic kidney and liver diseases. J Am Soc Nephrol.

[8] Connor A, Weston C, Dick C, Taylor JE (2009). Autosomal dominant polycystic kidney disease complicating renal ectopia and managed with renal transplantation. Clin Kidney J.

[9] Gupta M (2014). Ectopic autosomal dominant polycystic kidney disease (ADPKD)—an extremely rare radiological finding. Open J Radiol.

[10] Chen DP, Ma YY, Mao ZG, Mei CL (2010). Ectopic (pelvic) autosomal dominant polycystic kidney disease. Intern Med.

[11] Solak Y, Biyik Z, Gaipov A, Ozbek O, Tonbul HZ (2012). Ectopic, polycystic and stoned: pelvic kidney in a patient with autosomal dominant polycystic kidney disease. Am J Med Sci.

[12] Symeonidis A, Tsikopoulos I, Symeonidis EN, Tsifountoudis I, Michailidis A, Tsantila I, Gkekas C, Georgiadis C, Malioris A, Papathanasiou M (2022). More than meets the eye: a case of synchronous ipsilateral clear cell renal cell carcinoma and urothelial carcinoma of the pelvicalyceal system and literature review. Acta Biomed.

